# Supplementary material for: Comparative genomics provides new insights into the diversity, physiology, and sexuality of the only industrially exploited tremellomycete: Phaffia rhodozyma
Source: BMC Genomics. 2016 Nov 9;17:901. doi: 10.1186/s12864-016-3244-7 (PMC5103461; doi:10.1186/s12864-016-3244-7)
Supplement: Additional file 6: — List of orphan genes with links to PFAM (related to Additional file 1: Table S1). (ZIP 1428 kb) [file 12864_2016_3244_MOESM6_ESM.zip › PFAM_UNIQUE/G03726_P.html]

#                                                                            --- full sequence --- -------------- this domain -------------   hmm coord   ali coord   env coord
  
# target name        accession   tlen query name           accession   qlen   E-value  score  bias   #  of  c-Evalue  i-Evalue  score  bias  from    to  from    to  from    to  acc description of target
  
#------------------- ---------- ----- -------------------- ---------- ----- --------- ------ ----- --- --- --------- --------- ------ ----- ----- ----- ----- ----- ----- ----- ---- ---------------------
  
G03726\_P             -            196 DUF1125              PF06563.6     55      0.06   11.7   0.6   1   1   2.6e-05      0.16   10.4   0.5    16    42    92   122    88   133 0.77 - Protein of unknown function (DUF1125)  
G03726\_P             -            196 DUF4557              PF15101.1    212      0.01   14.4   0.9   1   2   6.8e-06     0.014   13.9   0.3     5    38    13    45    10    56 0.85 - Domain of unknown function (DUF4557)  
G03726\_P             -            196 DUF4557              PF15101.1    212      0.01   14.4   0.9   2   2       1.1   2.1e+03   -3.0   0.0    52    61   149   158   129   186 0.62 - Domain of unknown function (DUF4557)
